# QuickStats

**Published:** 2014-10-17

**Authors:** FROM THE NATIONAL CENTER FOR HEALTH STATISTICS

**Figure:**
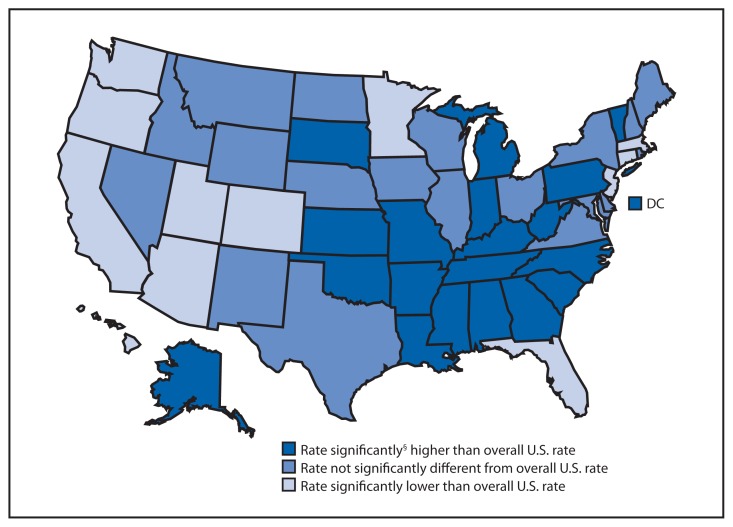
Age-Adjusted Rates* of Death from Fire or Flames,^†^ by State — National Vital Statistics System, United States, 2007–2011 * Age adjusted, per 100,000 standard population. ^†^ Based on International Classification of Diseases, 10th Revision codes X00-X09, X76, X97, and Y26, which include deaths from all intents (i.e., unintentional, suicide, homicide, and undetermined). ^§^ To identify state rates that were significantly higher or lower than the overall U.S. rate of 1.0 deaths per 100,000 population, differences between the U.S. and state estimates were evaluated using two-sided significance tests at the p<0.05 level.

During 2007–2011, age-adjusted rates for deaths from fire and flames varied widely by state, ranging from 0.3 per 100,000 population in Hawaii to 2.9 in Mississippi. In 18 states and the District of Columbia, the age-adjusted death rate was significantly higher than the overall U.S. rate of 1.0 per 100,000 population. Rates were higher than the U.S. rate in most of the southeastern states. In addition to Mississippi, the states with the highest death rates were Alaska (2.1), Alabama (2.0), Arkansas (2.0), and Oklahoma (2.0). The six states with the lowest death rates were Hawaii (0.3), Massachusetts (0.5), Arizona (0.6), California (0.6), Colorado (0.6), and Utah (0.6).

**Source:** National Vital Statistics System mortality data. Available at http://www.cdc.gov/nchs/deaths.htm. Rates of death from fire or flames, by state of residence, available at http://www.cdc.gov/nchs/pressroom/states/fire_flames.pdf.

**Reported by:** Holly Hedegaard, MD, hhedegaard@cdc.gov, 301-458-4460.

